# Generation of focusing ion beams by magnetized electron sheath acceleration

**DOI:** 10.1038/s41598-020-75915-8

**Published:** 2020-11-03

**Authors:** K. Weichman, J. J. Santos, S. Fujioka, T. Toncian, A. V. Arefiev

**Affiliations:** 1 Department of Mechanical and Aerospace Engineering, University of California at San Diego, La Jolla, CA 92093 USA; 2 University of Bordeaux, CNRS, CEA, CELIA, UMR 5107, 33405 Talence, France; 3 Institute of Laser Engineering, Osaka University, Osaka, 565-0871 Japan; 4 Institute for Radiation Physics, Helmholtz-Zentrum Dresden-Rossendorf e.V., 01328 Dresden, Germany; 5 Center for Energy Research, University of California at San Diego, La Jolla, CA 92037 USA

**Keywords:** Physics, Laser-produced plasmas

## Abstract

We present the first 3D fully kinetic simulations of laser driven sheath-based ion acceleration with a kilotesla-level applied magnetic field. The application of a strong magnetic field significantly and beneficially alters sheath based ion acceleration and creates two distinct stages in the acceleration process associated with the time-evolving magnetization of the hot electron sheath. The first stage delivers dramatically enhanced acceleration, and the second reverses the typical outward-directed topology of the sheath electric field into a focusing configuration. The net result is a focusing, magnetic field-directed ion source of multiple species with strongly enhanced energy and number. The predicted improvements in ion source characteristics are desirable for applications and suggest a route to experimentally confirm magnetization-related effects in the high energy density regime. We additionally perform a comparison between 2D and 3D simulation geometry, on which basis we predict the feasibility of observing magnetic field effects under experimentally relevant conditions.

## Introduction

Recent advances in all-optical magnetic field generation have made experimentally accessible new regimes of magnetized high energy density physics (HEDP) relevant to applications including inertial fusion energy^1–3^ and laboratory astrophysics^4–6^. In particular, the introduction of laser-driven coil targets^7–11^ capable of generating nanosecond-duration, hundreds of Tesla to kilotelsa-level magnetic fields over 100’s of microns at currently-existing large laser facilities including LFEX/GEKKO XII at ILE^7^, LULI^8,9^, and OMEGA^10,11^ introduces new possibilities in magnetized, relativistic laser-produced plasma. The understanding of the impact of strong magnetic fields on HEDP is rapidly evolving and has spurred research in areas including electron beam transport^12,13^, laser-produced magnetic reconnection^14^, and ion acceleration^15–17^.

In particular the ion acceleration induced by the expansion of a laser-heated electron sheath into vacuum^18,19^ presents an attractive platform for the study of magnetic field effects in laser-produced plasmas. Following its initial demonstration^20–22^, non-magnetized sheath-based ion acceleration has been extensively studied^23^, including in configurations compatible with experimental magnetic field generation platforms. Improvements in the ion source characteristics, including efforts to generate a focusing ion beam^24–26^ and increase the energy^27–29^, are additionally desirable for applications including isochoric heating^30^ and ion fast ignition^31^. It is therefore advantageous to elucidate the mechanism via which applied magnetic fields can beneficially alter sheath-based ion acceleration, particularly in the context of realistic magnetic field strengths.

Given the computational expense associated with 3D simulations, it would seem desirable to study the effect of the applied magnetic field in the context of 2D simulations. The limitations of using 2D simulations to represent 3D physics are well known in the target normal sheath acceleration (TNSA^32^) regime (i.e. without an applied magnetic field), with, for example, the conclusion that 2D simulations over-predict both the acceleration time and the maximum ion energy (e.g. Refs.^33,34^). However, the addition of the magnetic field as a new element in sheath-based ion acceleration requires re-evaluating the appropriateness of 2D simulations (such as those presented in Refs. ^9,15^) to study what is inherently a 3D phenomenon.

In this study, we present the first 3D fully kinetic simulations of sheath-based ion acceleration with a kilotesla-level applied magnetic field. We demonstrate that the magnetization of hot electrons creates a two-stage ion acceleration process consisting of enhanced energy gain and later focusing, resulting in a focusing, magnetic field-directed ion source of multiple species with strongly enhanced energy and number. We show that electron magnetization is tied to the balance of thermal to magnetic pressure in the hot electron sheath (plasma $\beta _e$), which changes over the course of the acceleration. The change in magnetization drives a fundamental change in the topology of the sheath electric field and reverses the usual outwardly diverging ion motion into focusing. We additionally find that the beneficial effects of the applied magnetic field are substantially downplayed in 2D simulations, on which basis we predict the feasibility of observing the acceleration mechanism we describe under experimentally relevant conditions.

## Results

We simulate a relativistically intense laser pulse interacting with the preplasma in front of an opaque plastic target with and without an applied magnetic field in 2D and 3D using the particle-in-cell code EPOCH^35^ (see “Methods”). The magnetic field strength and laser spot size were chosen to make 3D simulations tractable below machine-scale, which necessitated a 2000 T magnetic field. We also investigate the ability of 2D simulations to reproduce the magnetic field benefits observed in 3D at 2000 T. Following this analysis, we conduct additional 2D simulations with a 400 T field and larger laser spot to probe the relevance of the ion acceleration process we observe in 3D under experimentally realizable conditions. Unless explicitly stated, all simulation results were obtained from 3D simulations.

**Figure 1:**
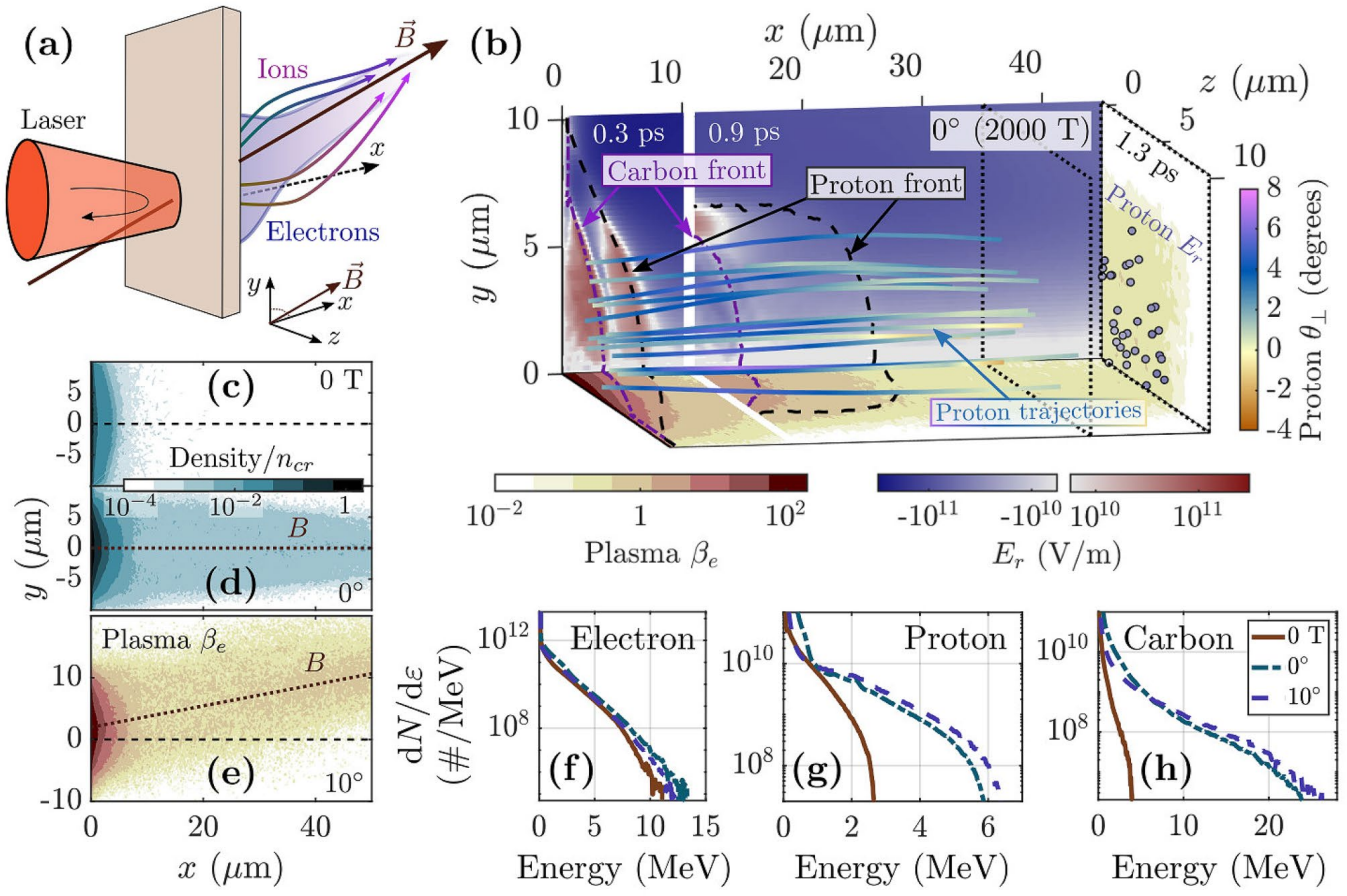
Ion acceleration with a strong applied magnetic field. (**a**) Schematic of simulation setup and ion acceleration. (**b**) Illustration of two-stage acceleration process for target-normal 2000 T magnetic field: (top) radial electric field, (bottom, right side) plasma $\beta _e$, and (trajectories) $\theta _\perp$, the angle the proton momentum makes with the *x*-axis, plotted along the trajectories of randomly selected protons. The protons shown have final energy above 4 MeV. Dashed lines indicate the proton and carbon ion front locations. The 3 times shown represent the first stage of the acceleration (0.3 ps), the beginning of the second stage (0.9 ps), and late into the second stage (1.3 ps). The slice in $\beta _e$ at $t=1.3~ \hbox {ps}$ corresponds to $x=35\;\upmu \hbox {m}$ (dotted plane). (**c**,**d**) Electron density at $t=0.3~ \hbox {ps}$ for (**c**) TNSA (no magnetic field) and (**d**) 2000 T target normal magnetic field. (**e**) Plasma $\beta _e$ at $t=0.3~ \hbox {ps}$ calculated from the density with 2000 T magnetic field directed at $10^\circ$ in the $x-y$ plane. (**f**) Energy spectra for electrons at $t=0.3~ \hbox {ps}$. (**g**,**h**) Final energy spectra for (**g**) protons and (**h**) carbon ions. (**g**,**h**) capture all ions with $p_x>0$.

The simulation setup is shown schematically in Fig. 1a. The laser pulse is linearly *y*-polarized, propagates in the *x* (target normal) direction, and is spatially and temporally Gaussian with $3~\upmu \hbox {m}$ FWHM spot size and 150 fs FWHM duration (both given in terms of the intensity). The wavelength is $1.06~\upmu \hbox {m}$ and the the peak intensity is $2\times 10^{19}~ {\hbox {W/cm}}^2$. The plastic (CH) target is $5~\upmu \hbox {m}$ thick with a $1.5\;\upmu \hbox {m}$ preplasma scale length. We apply a static uniform magnetic field of $B_0 = 2000~ \hbox {T}$ at either target normal incidence ($0^\circ$, $B_x = B_0$) or angled upward at $10^\circ$ in the *x*-*y* plane. For convenience, $t=0$ denotes the time when the peak of the laser pulse would impact the front target surface and $x=0$ is the location of the rear surface. See “Methods” for additional details of the simulation setup.

### Magnetic field-associated benefits to ion acceleration

Sheath-based ion acceleration is the transfer of electron thermal energy to ion energy mediated by a quasi-static electric field. When the laser interacts with the front surface preplasma, it generates a population of hot electrons. These electrons stream through the target and establish a sheath field on the rear surface, which then accelerates ions.

We find that the applied magnetic field has negligible impact on the laser production of hot electrons and does not substantially alter the laser-produced electron energy or angular spectrum (Fig. 1f). In our cases, the applied magnetic field is weak compared to the peak laser magnetic field and the electron gyro-frequency is low compared to the laser frequency, which precludes the resonant heating effect observed at substantially higher magnetic field strength^16,17,36^.

Although there is no apparent difference in the laser-produced hot electrons, we find that the accelerated ion energy and number, especially for the heavier ion species, are substantially enhanced by the application of the 2000 T field (Fig. 1g,h). This enhancement occurs because the magnetic field restricts the transverse spread of hot electrons within the target, enhancing the sheath electric field. Guiding of electrons within a target by a strong magnetic field has also been observed experimentally (for example, in Ref.^13^).

The guiding effect of the magnetic field is expected to be important when the magnetic field is able to substantially affect the transverse spread of hot electrons. For our simulation parameters, the laser spot size is comparable to the hot electron Larmor radius $\rho _L = cp_\perp /eB$, where we estimate $c p_\perp \sim T$ by the slope temperature $T \approx 0.8~\hbox {MeV}$ ($\text{ e}^{-\varepsilon /T}$ fit). The magnetic field reduces the hot electron transport across field lines and thereby increases the sheath electron density (e.g. Fig. 1c versus Fig. 1d) and the accelerating electric field, which increases the accelerated ion energy and number.

We additionally find that the magnetic field fundamentally changes the electric field configuration of the sheath *through the magnetization of hot electrons*, resulting in high energy ions which are (1) magnetic field-directed (the angular spectrum peaks along the field direction), and (2) magnetic field-focusing (coming to a focus along the field line).

### Role of electron magnetization in the sheath

Qualitatively, electrons are magnetized when the magnetic force dominates the electric force perpendicular to the field lines, i.e.

1 $$\begin{aligned} \left| e{\mathbf {v}}\times {\mathbf {B}}\right| /c > \left| e\mathbf {E_\perp }\right| , \end{aligned}$$

which requires at a minimum $B>E_\perp$. We estimate the sheath electric field generated by hot electrons as

2 $$\begin{aligned} E_\perp \approx 4 \pi |e| n_e \lambda _{De} = \sqrt{4 \pi n_e T}, \end{aligned}$$

where $\lambda _{De} \equiv \sqrt{T/4\pi e^2 n_e}$ is the hot electron Debye length corresponding to the local electron density $n_e$. We estimate $B \approx B_0$ for the magnetic field (the diamagnetic effect does not change the order of magnitude of *B*).

The relative strength of the electric and magnetic fields in the sheath is therefore approximately

3 $$\begin{aligned} E_\perp /B \sim \rho _L/\lambda _{De} \sim \sqrt{\beta _e}, \end{aligned}$$

where $\beta _e \equiv 8 \pi n_e T/B^2$ is the ratio of thermal to magnetic pressure. Thus we monitor $\beta _e$, which is associated with the collective processes of sheath formation and magnetic pressure, to infer the electron magnetization in the sheath.

**Figure 2:**
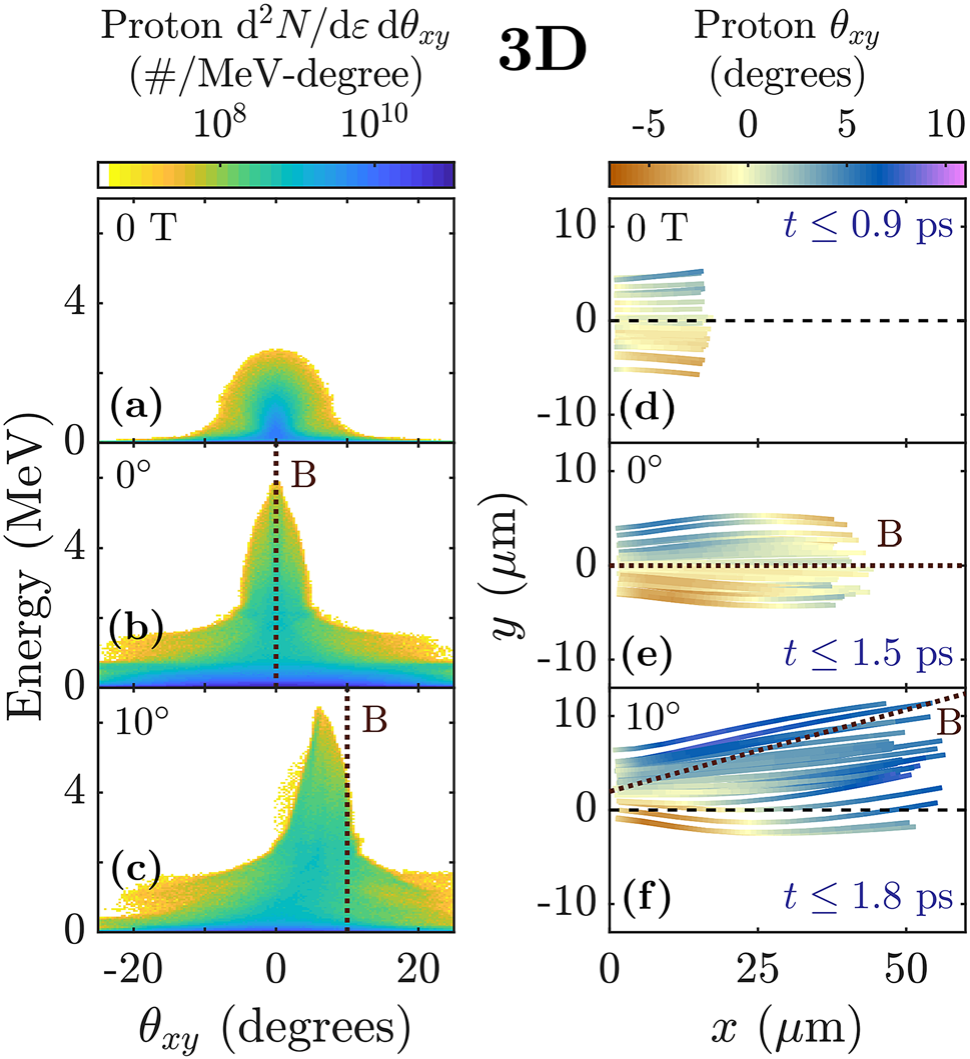
Ion focusing and deflection with applied magnetic field. (**a**–**c**) Angularly resolved proton energy spectrum corresponding to the cases shown in Fig. 1g. (**d**–**f**) $\theta _{xy}$, the angle between the proton’s forward momentum ($p_x$) and $p_y$, plotted along the trajectories of randomly selected protons. The protons shown have final energy above 2 MeV (0 T case) or 4 MeV (2000 T cases). (**a**,**d**) TNSA (no magnetic field). (**b**,**e**) Target normal 2000 T magnetic field. (**c**,**f**) 2000 T magnetic field directed at $10^\circ$ in the $x-y$ plane.

Close to the target surface and during the initial stage of acceleration, ions see an electron population with $\beta _e \gg 1$. During this stage, the ions quickly gain the majority of their final energy, which is enhanced by the addition of the magnetic field. However, there is no change to the electric field direction relative to a typical unmagnetized sheath.

**Figure 3:**
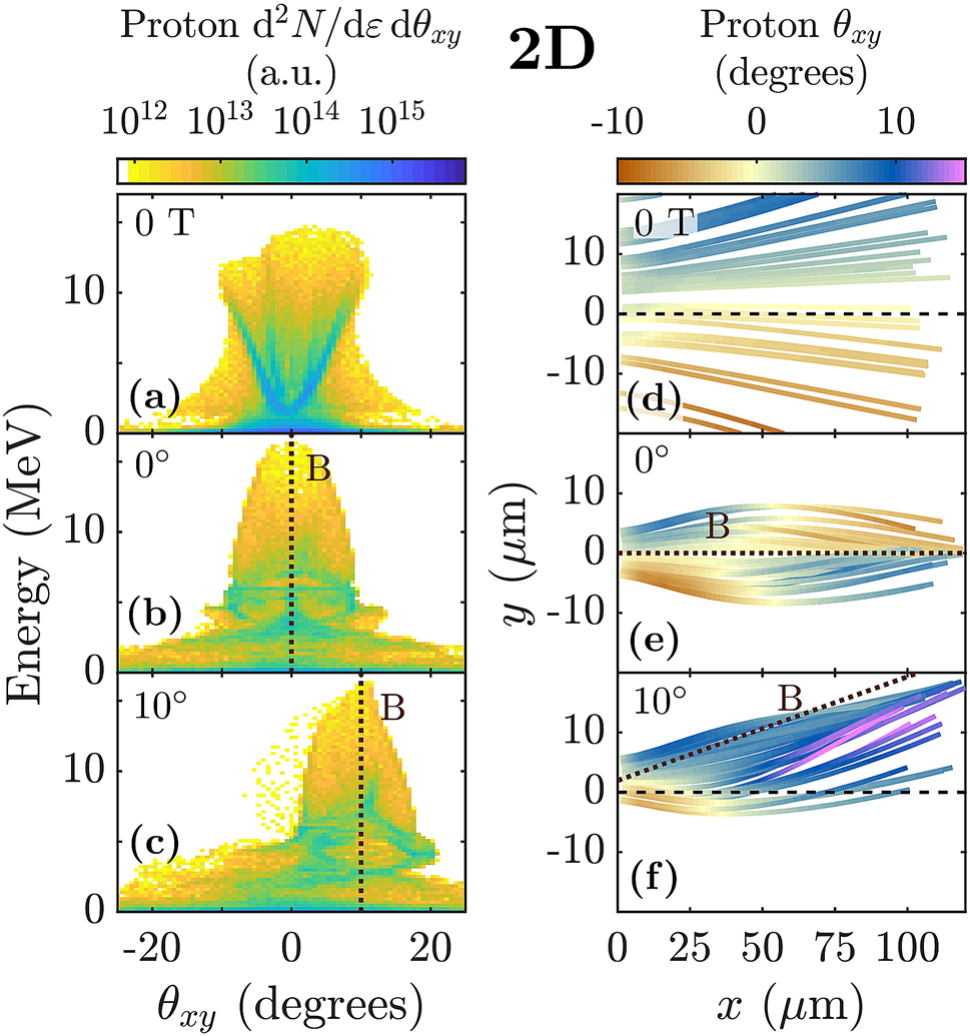
Ion focusing and deflection in 2D simulations. (**a**–**c**) Angularly resolved proton energy spectrum. (**d**–**f**) $\theta _{xy}$, the angle between the proton’s forward momentum ($p_x$) and $p_y$, plotted along the trajectories of randomly selected protons. The protons shown have final energy above 10 MeV. (**a**,**d**) TNSA (no magnetic field). (**b**,**e**) Target normal 2000 T magnetic field. (**c**,**f**) 2000 T magnetic field directed at $10^\circ$ in the $x-y$ plane. The angularly integrated energy spectra for the cases in (**a**,**b**) are shown in Fig. 4a.

Farther from the target surface and during the second stage of acceleration, high energy protons and carbon ions encounter an electron population with $\beta _e < 1$. This population is magnetic field-following (i.e. magnetized), which we demonstrate directly by tilting the magnetic field by $10^\circ$ in the *x*-*y* plane, e.g. in Fig. 1e. We find that the net deflection of electrons from the target-normal direction in the $10^\circ$ case causes the high energy ion population to be deflected as well (Fig. 2c,f). While the protons are still in the process of deflecting toward the field lines at the end of our 3D simulation, 2D simulations demonstrate that the high energy ion population becomes fully magnetic field-directed (Fig. 3c).

The magnetization of hot electrons additionally induces ion focusing about the magnetic field lines (e.g. Fig. 2e). The target-transverse, outward directed electric field experienced by ions during the initial, unmagnetized stage of the acceleration ($t\lesssim 0.5~ \hbox {ps}$, e.g. $x<10 \, \upmu \hbox {m}$ in Fig. 1b) results from the higher mobility of electrons than ions. The relative mobility of the hot electron population allows them to expand past the ions in both the target-normal and target-transverse directions and creates an initial outward expansion of the ions which is similar to the case with no magnetic field ($x\lesssim 10 \;\upmu \hbox {m}$ in Fig. 2d–f).

In contrast, in the magnetized sheath, the magnetic pressure exceeds the electron thermal pressure and the electrons become less mobile than the ions in the magnetic field-transverse direction, reversing the transverse sheath configuration. We find that as ions pass $\beta _e \lesssim 0.5$, the electric field becomes magnetic field-focusing ($t\gtrsim 0.5~ \hbox {ps}$, e.g. $x>10 \, \upmu \hbox {m}$ in Fig. 1b). This focusing effect persists over a long time and visibly pulls high energy ions toward the field lines (e.g. Figs. 1b and 2e,f). It also reduces the angular spread of high energy ions relative to the 0 T case (Fig. 2a–c).

This sheath-field-reversal induced focusing may also help explain astrophysical jet formation in an axial magnetic field. Jet formation has previously been studied in sub-100 T level magnetic fields, where several mechanisms have been proposed to explain ion collimation, including shocks^5^, gradients in magnetic pressure^37^, and pinching^38^. While these studies employed magnetohydrodynamic modeling due to the relatively large spatial ($\sim$mm) and time ($\sim$ ns) scales involved, in our study the high magnetic field and short laser pulse duration create spatial ($\sim 100\;\upmu \hbox {m}$) and time ($\sim$ ps) scales conducive to 3D kinetic modeling. As a result, we are able to separate the dynamics of the fully kinetic electrons from the ions. This approach has already enabled the discovery of novel astrophysically relevant ion acceleration-related phenomena under other conditions, for example in the colliding flows considered in Ref. ^6^. In our regime of relativistic intensity short pulse lasers and kilotesla-level magnetic fields, ion focusing is mediated by the ions transversely overshooting magnetized electrons from the high energy tail of the distribution and requires a fully kinetic treatment in lieu of single fluid magnetohydrodynamics.

### Necessity of 3D simulations

Although we observe the magnetic field-deflecting and magnetic field-focusing effects of electron magnetization in 2D simulations (Fig. 3), we find that 3D simulations are required to accurately capture the benefits of adding the magnetic field. This may be due in part to fundamental differences in physical processes which affect the strength of the sheath electric field in 2D versus 3D geometry. First, in 3D the hot electron sheath expands in two transverse directions, while in 2D it only expands in one, meaning the accelerating electric field drops less in 2D than in 3D for expansion over the same distance. Second, in 3D the electrostatic potential well created by charge separation has finite depth and allows sufficiently hot electrons to carry kinetic energy out of the system, while in 2D, the electrostatic potential does not converge as the hot electrons move away from the ions. As a result, in 2D even very hot electrons can transfer their full kinetic energy into sheath potential energy, while in 3D some of this energy would be lost. The effect of these differences can be seen even in TNSA (no magnetic field), where it is well-known that 2D simulations over-predict the ion energy (see, for instance the difference in peak energy in Figs. 2a and 3a).

**Figure 4:**
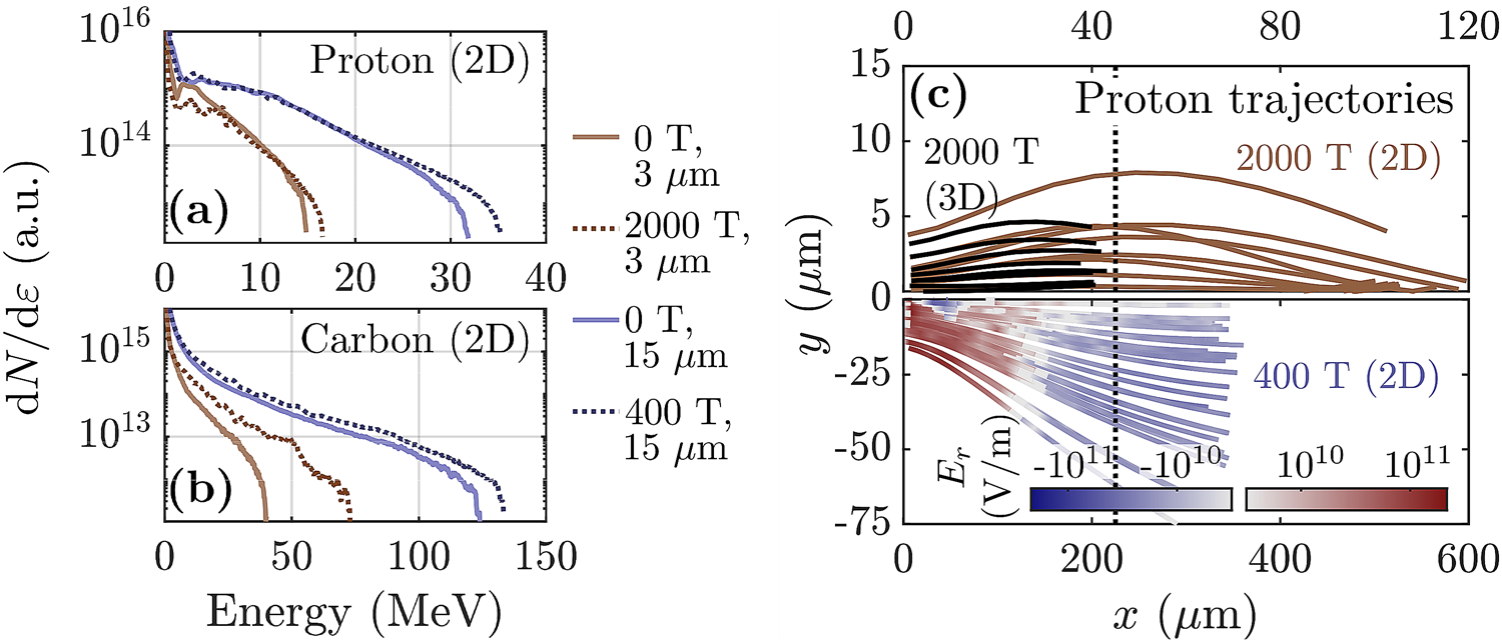
Ion acceleration in 2D simulations with target normal magnetic field and comparison with 3D. (**a**,**b**) Energy spectra in 2D simulations for 2000 T case ($3\;\upmu \hbox {m}$ focal spot) and 400 T case ($15\;\upmu \hbox {m}$ focal spot) for (**a**) protons, and (**b**) carbon ions. (**c**, top) 2000 T: comparison of proton trajectories in 2D and 3D. (**c**, bottom) 400 T: radial electric field experienced by randomly selected protons, plotted along their trajectories. Dotted lines in (**c**) denote (top) $x=45 ~ \upmu \hbox {m}$, roughly where $p_y$ changes sign in the 2000 T 2D case, and (bottom) $x=5\times 45=225 ~ \upmu \hbox {m}$. The protons shown have final energy above 4 MeV (3D case), 10 MeV (2D, 2000 T), or 25 MeV (2D, 400 T).

The addition of a sufficiently strong magnetic field modifies both the transverse expansion of the hot electron sheath and the behavior of the (now magnetized) electrons escaping the potential well, and clearly degrades the fidelity of 2D simulations. 2D geometry also fails to capture the 3D nature of cyclotron orbits. In a series of otherwise identical 2D simulations (same magnetic field strength, laser spot size, etc as the 3D cases), we observe that 2D simulations downplay the beneficial effects of the applied magnetic field. 2D simulations fail to reproduce the substantial energy enhancement observed in 3D simulations, e.g. the factor of 2 and 5 increases in the peak ion energy shown for proton and carbon ions in Fig. 1g,h, respectively. Instead, 2D simulations predict almost no enhancement in the peak proton energy and only a moderate increase in the peak carbon energy (brown lines in Fig. 4a,b). Additionally, we find that 2D simulations substantially over-predict the distances ions must propagate to be deflected towards the magnetic field lines and subsequently focused (e.g. Fig. 4c, top).

When 3D simulations are not tractable, for example at lower magnetic field strength, we can leverage the property that 2D simulations downplay the effects of the applied magnetic field to predict whether the magnetic field can still beneficially impact ion acceleration. Figure 4c, bottom shows the transverse component of the electric field experienced by high energy protons in a 2D simulation where we have decreased the magnetic field strength and increased the laser spot size by a factor of 5 ($B_x = 400~ \hbox {T}$, $15~\upmu \hbox {m}$ FWHM; keeping the ratio between the spot size and the Larmor radius roughly fixed). The transition from radially outward to radially inward electric field associated with the electron magnetization occurs later and the focal length is longer in the 400 T case than the 2000 T case, even when the distances are scaled by a factor of 5 (as in the visual comparison between the top and bottom panels of Fig. 4c). However, the transition to a focusing electric field still clearly occurs and the focusing field persists for long enough to visibly alter the direction of the ion momenta, on which basis we expect the benefits of adding the magnetic field to be observable in 3D at experimentally relevant field strengths.

## Discussion

In summary, the net result of adding a strong magnetic field is a magnetic field-directed, magnetic field-focusing ion source of multiple species with enhanced energy and number. The ion acceleration process features a fundamental change in the sheath dynamics mediated by the electron magnetization and occurs in two stages, an initial target normal stage with high energy gain and high divergence driven by electrons which are unmagnetized in the sheath but transversely confined through magnetization in the target, followed by a subsequent stage of ion deflection and focusing in the magnetic field direction driven by magnetized electrons. We term this two stage ion acceleration process magnetized electron sheath acceleration (MESA). We have additionally demonstrated that the benefits of adding the magnetic field are downplayed in 2D simulations, on which basis we predict the relevance of MESA under experimentally relevant conditions.

## Methods

We simulate a relativistically intense laser pulse interacting with the preplasma in front of an opaque plastic (CH) target with and without an applied magnetic field in 2D and 3D geometry using the open source particle-in-cell code EPOCH^35^. The laser pulse has a wavelength of $1.06~\upmu \hbox {m}$, is spatially and temporally Gaussian with a 150 fs FWHM duration and a $3~\upmu \hbox {m}$ FWHM spot size (both given in terms of the intensity), and has a peak intensity of $2\times 10^{19}~{\hbox {W/cm}}^2$. The preplasma has an exponential profile with a scale length (1/*e* density falloff) of $1.5~\upmu \hbox {m}$. This preplasma scale length was chosen to deliver robust TNSA-dominated acceleration in the case with no applied magnetic field. We model the target as a $5~\upmu \hbox {m}$ thick slab of fully ionized proton and carbon plasma with electron number density $n_e = 70\, n_{cr}$, where $n_{cr} \equiv m_e \omega _0^2/4\pi e^2$ is the critical density associated with the reflection of a laser pulse with frequency $\omega _0$. The plasma is initialized cold. The laser is linearly polarized in the *y*-plane, propagates in the *x*-direction, and is focused onto the front target surface. We apply a static uniform magnetic field of $B_0 = 2000~ \hbox {T}$ at either target normal incidence ($0^\circ$, $B_x = B_0$) or angled upward at $10^\circ$ in the *x*-*y* plane.

In 3D, the simulation domain is $90\times 37\times 24 ~ \upmu \hbox {m}$ for the largest simulation, which we resolve with 30 cells/$\upmu \hbox {m}$ in *x* and 20 cells/$\upmu \hbox {m}$ in *y* and *z*. The target rear surface, which we have defined as $x=0$, is located $25~\upmu \hbox {m}$ from the simulation boundary. Electrons, protons, and carbon ions are represented by 10, 5, and 5 cubic B-spline macroparticles per cell through most of the domain, with 20 macroparticles per cell for protons and carbon ions within $0.5~\upmu \hbox {m}$ of the target rear surface to better resolve the ion spectra. The use of high order particle shape such as cubic B-spline has been demonstrated to mitigate numerical heating (for example, in EPOCH^35^), and in our simulations delivers robust energy conservation. The simulation is run until the highest energy protons begin to leave the simulation box. The field and density quantities shown in figures were temporally averaged over 5 laser cycles (18 fs) and spatially averaged over $0.3~\upmu \hbox {m}$.

In the 2D simulations we conduct to compare with the 3D cases, the simulation domain is $170\times 80 ~ \upmu \hbox {m}$ for the ordinary TNSA ($B_0 = 0$) case. In the cases with a $15\;\upmu \hbox {m}$ spot size, simulation domain is $550\times 200 ~ \upmu \hbox {m}$ for the largest simulation ($B_0=400~ \hbox {T}$), which we resolve with 30 cells/$\upmu \hbox {m}$ in both *x* and *y*. In this simulation, the target rear surface, which we have defined as $x=0$, is located $55~\upmu \hbox {m}$ from the simulation boundary. Electrons, protons, and carbon ions are represented by 50, 25, and 25 cubic B-spline macroparticles per cell through most of the domain, with 150 macroparticles per cell for protons and carbon ions within $0.5~\upmu \hbox {m}$ of the target rear surface. The simulation cost for the 400 T case was approximately the same as the 3D simulations.

## Data Availability

The datasets generated and analysed during the current study are available from the corresponding author on reasonable request.

## Code availability

The particle-in-cell simulation code EPOCH^35^ used during the current study is freely available from the University of Warwick (https://cfsa-pmw.warwick.ac.uk/EPOCH/epoch).
